# Flexural Strength Characteristics of Fiber-Reinforced Cemented Soil

**DOI:** 10.3390/ma16114185

**Published:** 2023-06-04

**Authors:** Gabriel Orquizas Mattielo Pedroso, Ricardo Domingos dos Santos Junior, Jefferson Lins da Silva, Mariana Ferreira Benessiuti Motta, Emerson Felipe Félix

**Affiliations:** 1 Department of Civil Engineering, Faculty of Engineering and Sciences, São Paulo State University, Guaratingueta 12516-410, Brazilemerson.felix@unesp.br (E.F.F.); 2 Department of Transport Engineering, São Carlos School of Engineering, University of São Paulo, Sao Carlos 13566-590, Brazil

**Keywords:** cemented soil, flexural performance, polypropylene fibers, steel fibers, pavement

## Abstract

This work deals with the flexural performance of a soil-cement for pavement reinforced by polypropylene and steel fibers, and the main purpose is to evaluate the effect of different curing times. In this sense, three different curing times were employed to investigate the influence of fibers on the material’s behavior at varying levels of strength and stiffness as the matrix became increasingly rigid. An experimental program was developed to analyze the effects of incorporating different fibers in a cemented matrix for pavement applications. Polypropylene and steel fibers were used at 0.5/1.0/1.5% fractions by volume for three different curing times (3/7/28 days) to assess the fiber effect in the cemented soil (CS) matrices throughout time. An evaluation of the material performance was carried out using the 4-Point Flexural Test. The results show that steel fibers with 1.0% content improved by approximately 20% in terms of initial strength and peak strength at small deflections without interfering the flexural static modulus of the material. The polypropylene fiber mixtures had better performance in terms of ductility index reaching values varying from 50 to 120, an increase of approximately 40% in residual strength, and improved cracking control at large deflections. The current study shows that fibers significantly affect the mechanical performance of CSF. Thus, the overall performance presented in this study is useful for selecting the most suitable fiber type corresponding to the different mechanisms as a function of curing time.

## 1. Introduction

With a lack of good-quality natural resources and the increasing cost of construction materials, sustainable techniques and materials that adapt to the location have been increasingly incorporated into highway and airway pavement projects. In the past decades, chemical soil stabilization has been widely used in pavement structure. This technique combines the use of local soil with cement to provide a strong material, which, when used in layers of the pavement, can support traffic loads [1,2,3,4,5,6].

For example, Consoli et al. [3] quantify the impact of cement quantity, porosity, and voids/cement ratio on the assessment of split tensile strength in sandy soil when reinforced and non-reinforced with fibers. A comprehensive series of split tensile tests were conducted to investigate this relationship. In summary, the results highlight the influence of cement quantity, porosity, and voids/cement ratio on split tensile strength in both fiber-reinforced and non-reinforced cemented soil. The findings demonstrate the positive effect of fiber reinforcement on split tensile strength and provide valuable insights into the relationships between these parameters for the effective evaluation of the studied soil mixture.

Even though the use of local soil with cement provides a strong material, CS presents brittle behavior at failure, which means that, once the yield stress is exceeded, the material suddenly loses all or most of its initial support capacity. Furthermore, during their useful life, cemented bases on highways are frequently subjected to repeated loads that lead to failure and crack formation due to fatigue mechanisms [7,8].

One solution being studied to reduce these undesired behaviors is incorporating fibers into CS, which has been an efficient technique for improving the material’s mechanical properties. The first studies in the area began with McGown et al. [9], who divided fiber reinforcement into two main categories: inextensible and extensible. The first type includes high modulus of elasticity materials such as metal bars and strips, while the latter incorporates the relatively low modulus of elasticity of natural, synthetic, and glass fibers. According to previous studies [10,11,12,13,14], fibers do not prevent crack formation, but they act directly to control propagation with the cemented mass, benefiting the mechanical properties of the pre- and post-rupture states.

Sukontasukkul and Jamsawang [12] investigate two types of fiber to produce fiber-reinforced soil-cement: polypropylene and steel fibers at three different volume fractions of 0.5%, 0.75%, and 1.0%. To improve flexural strength and brittleness, a technique of mixing short fibers was introduced to the soil-cement mixture. The results pointed out notable enhancements in the flexural performance of the material when incorporating fibers into the mix, as evidenced by an increase in the equivalent flexural strength ratio and residual strength. Comparatively, polypropylene fibers demonstrate superior performance compared to steel fibers. Furthermore, as the volume fraction of fibers increases, there is a corresponding increase in toughness.

According to Jamsawang et al. [7], it is essential to consider that structural pavement layers are primarily subjected to tensile and flexural stresses rather than compressive stresses. In contrast, cement-treated soils possess significantly lower tensile and flexural strengths when compared to their compressive strengths. Moreover, when exposed to bending loads, cement-treated soils tend to exhibit brittle behavior, which is unfavorable for pavement materials. Ductile behavior is crucial for pavement materials to withstand the pressures of heavy traffic loads, prevent immediate failure, and effectively increase the lifespan of the pavement. Thus, flexural strength is significant in pavement design and is used to determine slab thickness ASTM D1635-12 [15].

Considering the purpose of improving the mechanical performance of soil-cemented elements, one modern method of soil reinforcement involves using continuous, flat geosynthetics, including geotextiles, geonets, geofabrics, and geomembranes [16]. However, it is important to note a specific limitation of this type of reinforcement. Li [17] points out that, while it does increase the soil strength in a particular direction, it also leads to the formation of a plane with reduced shear strength at the interface between the geosynthetics and the soil, compared to unreinforced soil.

An alternative approach to soil reinforcement involves using short, randomly oriented fibers within the soil. Li [17] argues that when fibers are uniformly mixed with the soil, they can provide an isotropic increase in the strength of the composite material without introducing planes of reduced shear strength. In addition, this type of reinforcement does not require considerations regarding anchoring design.

The behavior of fibers in the ground is compared to that of plant roots. The presence of fibers helps distribute shear stresses in the soil through inclusions with relatively high tensile strength. This behavior enhances soil stability and improves its mechanical properties [16].

Hejazi et al. [18] conducted a literature review exploring fibers’ properties, applications, and effects in reinforced soils. The authors highlight the importance of thorough analysis and optimization of fiber properties, such as diameter, length, surface texture, and reinforcement mechanism, for effective soil reinforcement. Soil reinforcement can be categorized into two groups based on their modulus of elasticity, which determines their performance characteristics in the ground medium. The first group consists of high modulus of elasticity (rigid) materials, including steel strips, glass fibers, and basalt fibers. These materials are primarily used to strengthen the soil and restrict internal and boundary deformations. The second group comprises natural and synthetic fibers and geosynthetics with a low modulus of elasticity (ductile). These materials contribute to soil reinforcement and offer operational properties in post-critical stress areas. This group includes synthetic fibers such as polypropylene (PP), polyester (most commonly PET), polyethylene (PE), glass fibers, nylon fibers, and polyvinyl alcohol (PVA) fibers. The advancement of plastic production technology has increased the interest in plastic-based fibers within the modern industry. These synthetic fibers provide additional options for soil reinforcement due to their various properties and characteristics.

Overall, Hejazi et al.’s [18] review underscores the importance of understanding and optimizing the properties of fibers used in reinforced soils, as well as differentiating between high modulus of elasticity materials for soil strength and low modulus of elasticity materials for post-critical stress properties. The inclusion of synthetic fibers has expanded the range of options available for soil reinforcement in contemporary applications.

In practical applications, steel and synthetic fibers such as polypropylene (PP) or nylon fibers are commonly adopted for reinforcing cement-treated soils. Each fiber type offers its advantages and disadvantages when used for reinforcing cemented soil. Using steel fibers improves the tensile strength of cemented soil elements, as well as ductility, durability, and fatigue resistance. However, they have a disadvantage regarding corrosion susceptibility and higher cost than PP fibers. The PP fibers have the advantage of crack propagation control, being cost-effective, and being lightweight and easily blendable with cement, ensuring a uniform distribution throughout the soil mixture. However, PP fibers have lower tensile strength than steel fibers and are more sensitive to temperature [16].

Including fibers in soil or soil-cement is a reinforcement method still incipient in the pavement area [7,19]. This technique is already used in cemented materials to control the cracks and prevent the material from a fragile and catastrophic failure [6,12,20,21,22]. Most of these studies conducted laboratory tests using flexible synthetic fibers [6,7,12,22,23,24,25,26] and steel fibers [7,12,23,26,27]. They reveal that the inclusion of the two fibers leads to significant improvements in the tensile and flexural strength of the cemented materials, increasing the toughness and the ability of the material to resist stresses even after cracking because the fibers serve as “bridges” mobilizing a wider mass of soil-cement, allowing a better redistribution of the tensions in the matrix [13,28].

In terms of the mechanical performance of fibrous composites, most studies focus on unconfined compression [24,27,29,30,31,32] and splitting tensile strength tests [3,8,33,34,35] due to the availability and familiarity with the apparatus. However, these tests do not reflect the stresses on the pavement [7,12,19]. In fact, pavement structures are subject to flexural loads that cause tensile stress and crack at the bottom of the cemented base layer [35,36,37]. Therefore, for a better characterization of cemented materials in the pavement, recent studies [36,38,39] have used the flexural test in mechanical performance due to its similarity with the stress condition produced in the field.

Despite this, the evaluation by the static flexural test using this standard offers an alternative fiber dosage for the material. Previous studies [6,7,12,21,27,38,40] recommend a fiber content that gives the material a deflection-hardening behavior, that is, after crack, it must support and even increase its strength, indicating that the fibers are sufficient to transfer loads of the same magnitude even after the crack, with a prevalence of multiple and small cracks instead of one big crack in the material.

Furthermore, comparing fibers is essential to select the most suitable fiber according to the design purpose. In flexural performance, a fiber that provides a deflection-hardening response in lower fiber content is considered more cost-effective and likely to be used in engineering applications [7,27,38,40]. Sukontasukkul and Jamsawang [12] compared the flexural performance of a soil-cement matrix at 20% of cement content with PP and steel fiber at 0.5–1.0% of fiber content. They concluded that the PP fiber contributed more significantly to post-peak strength, ductility, and toughness than steel fibers. Although a high cement content was used, the flexural strength reached values between 0.1 and 0.2 MPa, representing a poor bond between the soil and the cement particles in the matrix. In this case, deflection-hardening behavior was not achieved.

Most parts of previous studies focused on using only one type of fiber and finding the best fiber content for that specific matrix. The few papers that approach the two groups of fibers do not analyze the issue concerning that different fiber natures have different contributions due to the matrix’s changing strength and stiffness. Therefore, this research aims to compare the flexural performance of a soil-cement for pavement reinforced by polypropylene and steel fibers considering the action of different curing times. In this sense, three different curing times will be employed to investigate the influence of fibers on the material’s behavior at varying levels of strength and stiffness as the matrix becomes increasingly rigid. In this way, it is possible to identify which type and fiber content are the most suitable for the CS matrix and how the curing time affects the efficacy of the contribution of each fiber. The use of curing time as a factor is a novelty in pavement areas and aims to identify the fiber contribution along time, because it is believed that the results are more related to strength and stiffness reached by the matrix than the cement content utilized.

## 2. Materials and Methods

This section presents the experimental procedures and material utilized to investigate the flexural performance of a soil-cement for pavement reinforced by polypropylene and steel fibers.

### 2.1. Test Materials

A typical clayed sand soil (SC) classified as A2-4 according to the USCS and the HBR classification system from Brazil was utilized in this study. This soil is typically used for infrastructure construction in São Paulo, Brazil, and Table 1 provides the main geotechnical properties of the soil.

Ordinary Portland cement with the addition of slag classified as CP II E-32 by the Brazilian standard, corresponding to type I cement in ASTM C150/C159M-22 [41], was used as the curing agent. According to NBR 11578, this type of cement can have 6 to 34% slag in its composition, and its compression strength at 28 days must be greater than or equal to 32 MPa. The CS dosage was carried out following the Brazilian standard NBR 12253 [42]. The 6% cement by (dry weight of soil) was considered optimum and used to prepare all specimens for the current study.

**Figure 1:**
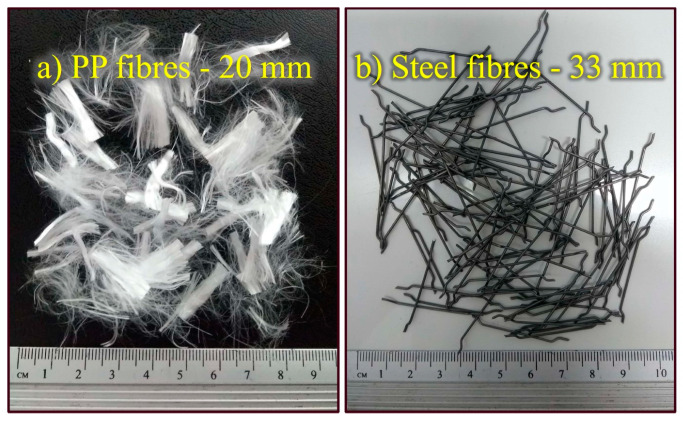
Fiber types: (**a**) PP fibers; (**b**) steel fibers.

In this study, 20 mm polypropylene and 33 mm steel fibers were used as reinforcement elements. These fibers are commercially available in large quantities in Brazil and can be used in future field tests or engineering projects. Furthermore, previous studies with similar PP fibers [3,43] and steel fibers [7,12] showed satisfactory results. Figure 1 shows the appearance of each fiber, and Table 2 summarizes their main physical and mechanical properties.

### 2.2. Sample Preparation

The clayed sand was dried using a convective oven for 24 h to obtain a minimal initial moisture content (varying from 0.2 to 0.5%) two days before mixing with the cement and compaction. The amount of 6% of cement in dry weight was incorporated into the soil and mixed for 3 min. Next, water was added to the CS and homogenized according to the optimum moisture content of 8.5%, as determined by intermediate Proctor tests, for an additional 3 min. Finally, the PP or steel fibers were manually mixed with the CS, a procedure recommended by Consoli et al. [3]. The amount of fiber used refers to the dry weight of soil plus cement.

A preliminary separation procedure was carried out before the mixing process of the PP fibers to avoid the aggregation problem. A similar process was used by Cristelo et al. [32], and it consisted of injecting compressed air into a bag containing the original fibers for 5 min.

The testing apparatus consists of a rectangular beam measuring 100 mm wide, 100 mm high, and 350 mm long. Each sample was dynamically compacted in three layers using a manual hammer with a rectangular base of 50 mm × 50 mm and a mass of 2.5 kg dropped from a height of 30.5 cm with 130 blows per layer. Each layer was scarified after compaction to improve the bond between layers. One day after the compaction process, the specimens were demolded and then wrapped in a plastic sheet to avoid moisture variation and were then cured inside a controlled room with a temperature of 23 °C ± 2 °C until the target curing time (3, 7 or 28 days).

The samples were considered acceptable for testing when they met the following criteria: dry density values between 99% and 101% determined by the intermediate Proctor test, moisture content varying ±0.5% from the optimum value obtained by the intermediate Proctor test, diameter tolerance between ±0.5 mm, and height tolerance between ±1.0 mm.

### 2.3. Experimental Program

The experimental program was divided into two parts. The first consisted of studying Proctor compaction at the intermediate energy of CSF to understand the effect of including fibers in compaction. In the second part, static flexural tests were carried out to evaluate the influence of the type of fiber, fiber content, and curing time on the mechanical behavior of the material. The results were obtained as the average value of three specimens. Therefore, the study variables of interest are:

- Type of fiber: since different natures of fiber can offer different contributions to mechanical behavior, this study focuses on the influence of fiber (polypropylene or steel fiber).
- Fiber content: It is essential to use different fiber amounts to check trends caused by the fiber content increase in the matrix. Therefore, to evaluate the fiber effect on flexural behavior, three percentages of fiber contents were used in the experiment: 0.5%, 1.0%, and 1.5%.
- Curing time: three curing times were used (3, 7, and 28 days) to find different trends in how each fiber influences strength and stiffness within the evolution of the matrix becoming stronger and more rigid.

### 2.4. Four-Point Static Flexural Test

**Figure 2:**
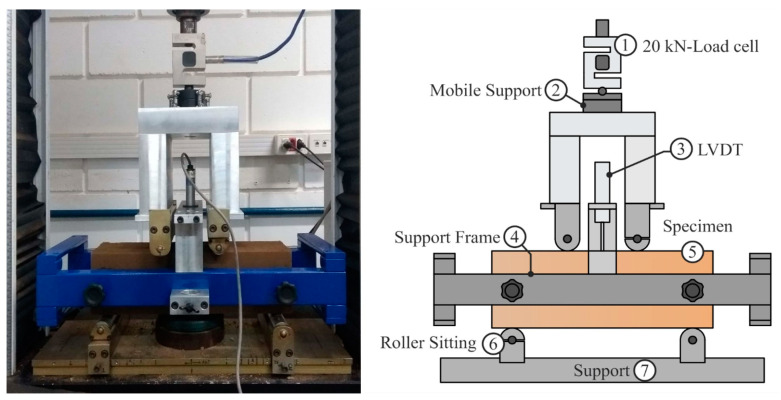
Flexural specimen and 4-Point Flexural Test set-up.

The specimens were submitted to the 4-point flexural test after the curing time. The test was performed according to the ASTM C1609/C1609-12 [44] in an Instron^®^ (Norwood, MA, USA) universal testing machine. The beams were centralized and aligned parallel to the rollers sitting support and the LVDT’s support frame. An external load cell with a capacity of 10 kN was used to measure the loading, while an LVDT was positioned in the center of the support to measure the deflections in the middle of the tested beam. The static flexural tests were carried out in a displacement-controlled mode, in which a deflection rate of 0.075 mm/min was used, as recommended by the standard, until deflection reached 3 mm. The geometry of the test specimen and the test setup for the flexural test are shown in Figure 2.

Regarding mechanical behavior, each tested material has a specific behavior concerning the load–deflection curve, which is governed by the fiber content in its matrix. The CS without fiber presents brittle behavior, the flexural strength drops to zero after the first cracking, and the fibrous composite can exhibit two distinct behaviors: deflection-hardening or deflection-softening [6,40]. The first is characterized by an increase in the load capacity after the first rupture and multiple cracks, while the second is identified by a drastic drop (single crack) in the load that can be followed by either a second increase or a continued decrease in strength [6,40].

The first point of analysis is the first cracking point, which is defined as the point that represents the transition from linearity to non-linearity behavior of a load–deflection curve. This point is known as the limit of proportionality (*LOP*). The next point is where the maximum load occurs after the *LOP*. This point is known as the modulus of rupture (*MOR*) and corresponds to the maximum load supported by the pull-out force of the fibers after the initial crack. Note that the *LOP* and *MOR* are coincident points for composites without fiber. Equation (1) was used to calculate the flexural stress, and the maximum load between *LOP* and *MOR* point was used to evaluate the peak strength of the specimen.

(1) $$f_{LOP}=\frac{P_{i}\times L}{{b\times d}^{2}}$$

where *P_i_* is the maximum applied load (N), *L* is the span length (mm), *b* is the width of the specimen (mm), and *d* is the depth of the specimen (mm).

Standard ASTM C1609-12 [44] recommends L/600 (corresponding to a net deflection equal to 1/600 of the span, 0.5 mm) and L/150 points to be evaluated in flexural behavior; L/300 point was additionally added to this study for a complete analysis. The load applied after these points corresponds to the residual strength.

For cemented base materials with or without fibers, stiffness is a key parameter in developing mechanistic analysis in the pavement. Therefore, to evaluate fiber effect on material stiffness and future stress–strain analysis, the static flexural modulus was determined from the stress–deflection relationships (secant modulus corresponding to 50% of MOR) by Equation (2).

(2) $$S_{m}=\frac{23PL^{3}}{108bd^{3}\delta }\left[1+\frac{216d^{2}(1+\nu )}{115L^{2}}\right]$$

where *S_m_* is the static flexural modulus (MPa), *P* is the load (N) for the corresponding deflection *δ* (mm), *ν* is the Poisson’s ratio of the stabilized material and *L* is the length between supporting rollers; w and h are the average width and height (mm), respectively. The value of *ν* was assumed to be 0.2.

In addition to flexural strength and the static flexural modulus, other parameters are commonly used to describe the flexural behavior of composites. In this study, the ductility index and toughness were evaluated to compare the flexural performance of the CSF.

According to Jamsawang et al. [7], the ductility index of a composite material is the ratio between the deflection at the modulus of rupture (*MOR*) and the first crack deflection (*LOP*). The higher the ratio, the more ductile the fibrous composite. Therefore, the ductility index (*DI*) can be defined from Equation (3).

(3) $$DI=\frac{\delta_{MOR}}{\delta_{LOP}}$$

Toughness is the energy absorption related to the area under the load–deflection curves at a specific point. According to Khattak et al. [2] and Sobhan et al. [45], higher energy absorption materials have higher fatigue failure strength, which is desired in pavement applications. In this study, the toughness was calculated using the area below the load–deflection curve until the determined deflection point (0.5, 1.0 and 2.0 mm).

## 3. Results and Discussion

### 3.1. Dry Unit Weight and Water Content

**Figure 3:**
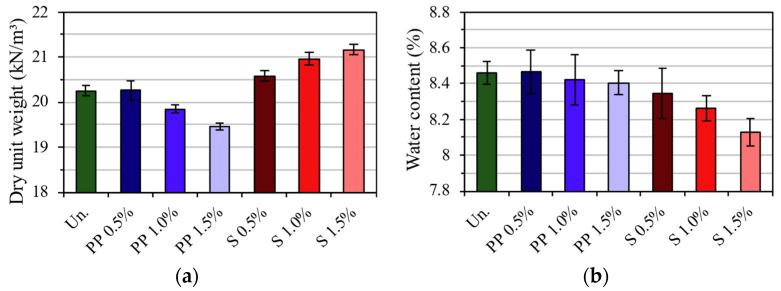
Control Parameters of the specimens: (**a**) dry unit weight; (**b**) water content.

The compaction for the flexural tests of the CS and CSF mixtures was performed on Proctor’s intermediate energy. The influence of fiber and fiber content on dry density and water content is shown in Figure 3.

Firstly, there were decreases of 0.04%, 2.03%, and 3.90% in the dry unit weight for the mixtures with PP fiber at the contents of 0.5, 1.0, and 1.5%, respectively. According to Onyejekwe & Ghataora [23] and Tran et al. [34], this tendency of higher PP fiber content resulting in dry unit weight reduction can be explained by the fact that flexible fibers absorb a part of the compaction energy.

The steel fiber mixtures had an increase of 1.61, 3.49, and 4.51% in dry unit weight for samples with fiber contents of 0.5, 1.0, and 1.5%, respectively. Although there is no research focusing on the steel fiber effect in mixtures with soil, these increases were expected since the volume of steel in the mix corresponds to a much larger mass than if the CS matrix occupied this volume.

The optimum water content was essentially the same for PP fiber since they do not absorb water and do not represent a significant percentage of the total mass of the mixture (approximately 0.75%). On the other hand, there was a slight decrease in the optimum water content due to the inclusion of steel fibers since the total mass of the mixture is significantly increased by the volume of steel fiber (approximately 6%).

### 3.2. Flexural Behavior of Load–Deflection Curves

**Figure 4:**
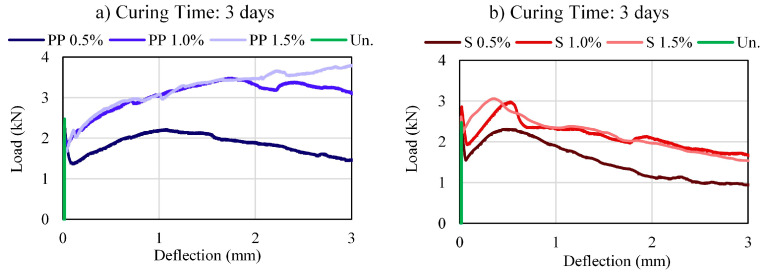
Load–deflection curves of the mixtures: (**a**) PP—3 days; (**b**) Steel—3 days.

**Figure 5:**
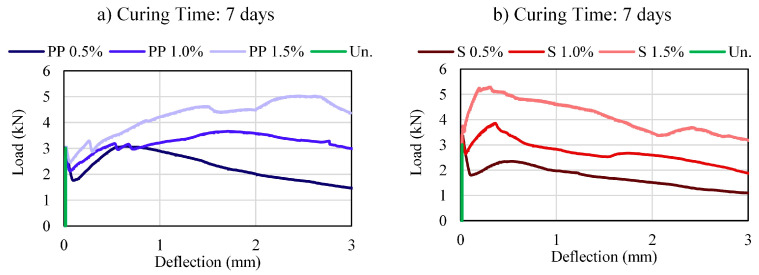
Load–deflection curves of the mixtures: (**a**) PP—7 days; (**b**) Steel—7 days.

**Figure 6:**
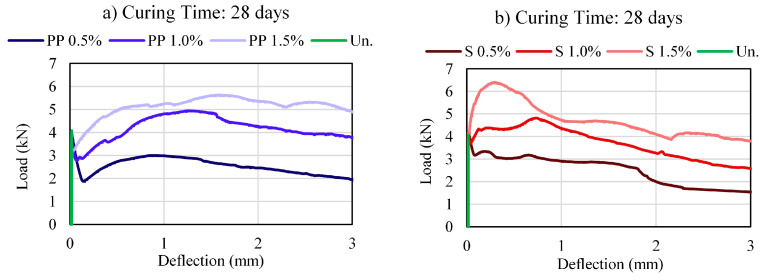
Load–deflection curves of the mixtures: (**a**) PP—28 days; (**b**) Steel—28 days.

Figure 4, Figure 5 and Figure 6 present the load–deflection curves for CS and CSF for different fiber types and contents. The figures clearly show how the type and fiber contents affect the load–deflection response, directly affecting load-carrying ability, deformability, ductility, and toughness.

The cemented soil without fiber showed brittle behavior, that is, the load is linearly increasing until it reaches its maximum and drops drastically to zero. The cemented soil with fiber samples presented LOP deflection close to those without fiber, indicating that the linear trend until the first cracks are not affected by the fiber. However, after this point, the load capacity of the fibers specimens continues to increase, exhibiting a deflection-hardening or softening behavior, characteristic of ductile materials and dependent on the fiber contents [38].

The deflection value at the MOR point is affected by the fiber type. Therefore, the PP fiber presented MOR deflections ranging from 1 mm to 3 mm, while the steel fiber had MOR deflection between 0–1 mm.

The post-cracking response had different behavior due to the fiber type. PP fiber maintains higher strengths close to their peak strength, while steel fiber has a more considerable loss in strength to higher deflections. Therefore, PP fibers are mobilized with more significant deformations than steel fibers.

The deflection hardening behavior of the composite can be enhanced by increasing the gap between the LOP and MOR and their corresponding deflections [36]. Regarding the load–deflection curve response, at 0.5% of fiber volume, both fiber mixtures showed a deflection-softening with a low load-carrying capacity after first cracking. The increase in fiber content contributes to a rise in the deflection-hardening behavior of the material, and for a complete deflection-hardening behavior, with an immediate rise in load after first cracking (LOP), 1.5% fiber content was necessary for both PP and steel fibers. In addition, it is noted that a curing time of 28 days was required to fully develop this characteristic, especially for steel fibers.

The increase in curing time reduces the load drop after the first crack (LOP) and may represent an increase in the bond strength and the contribution of the fiber in the matrix. Furthermore, the peak strength (MOR) deflection decreases in function of the curing time. In other words, less deformation is required to activate the pull-out forces at the fiber soil-cement interface with the increase in curing time.

### 3.3. LOP and Peak Strength

**Figure 7:**
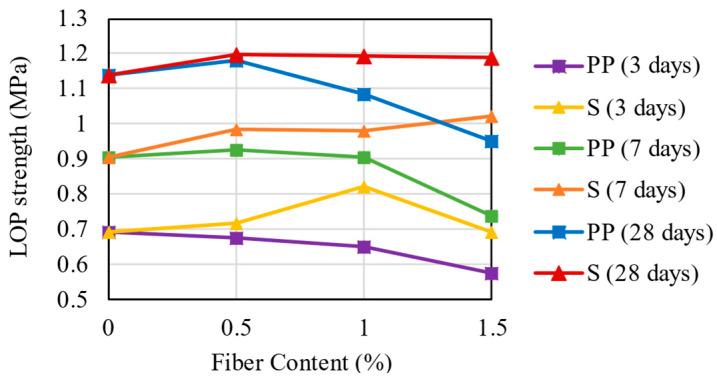
LOP Strength Results for the mixtures.

Figure 7 shows the effect of fiber type, fiber content, and curing time on the values of LOP. The increase in PP fiber content has a negligible impact on the LOP strength of the material. Two suppositions can explain this: the first relies on the decrease in dry unit weight, resulting in a specimen with less cement and fewer compacted particles in the mixture due to loss of compaction energy by the PP fibers. Moreover, the second is attributed to the difficulty in inserting the PP fibers in the matrix, which, in large quantities, can agglomerate, creating a barrier that hinders the adhesion between the CS particles. In other words, the CSF depends mainly on the strength of the CS matrix rather than the fiber cohesion [46].

Differently from the PP fiber, the steel fiber presents slightly higher LOP strength with the increase in its content. However, these values did not follow a specific trend, and the rate of increase in LOP is not significant, which implies that the steel fibers used in this study did not interfere with the LOP strength of the mixtures.

**Figure 8:**
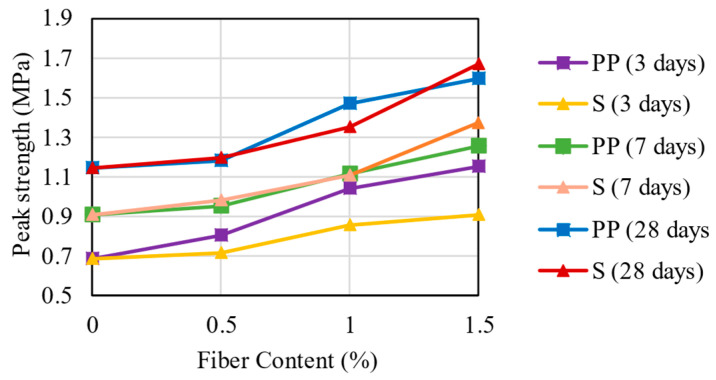
Peak Strength Results for the mixtures.

Figure 8 presents the average values of the peak strength, which is the higher value between MOR and LOP strength, for the mixtures with PP and steel fibers, considering the three different curing times. First, it is noted that all mixtures with fibers had higher peak strength than the CS matrix (0.58 MPa). At 3 days, the insertion of fibers in the contents of 0.5, 1.0, and 1.5% contributed to the increases of 16.5, 50.7, and 67.0% in the peak strength, respectively, for the PP fibers. On the other hand, the steel fibers had an increase of 3.8, 24.4, and 31.8% for the contents of 0.5, 1.0, and 1.5%, respectively. This result indicates that the rate of increase in peak strength of the PP fiber is higher in comparison with the steel fiber, which implies that PP fibers can contribute to the load faster at the same fiber content.

At 7 days, the contribution rate of PP fibers to peak strength decreased significantly (5.2/23.2/38.5%) compared to 3 days. In contrast, steel fibers maintained the contribution rate for the volumes of 0.5 and 1.0% (8.7 and 22.1%) and significantly increased for 1.5% of fiber content (51.8%). The increase in fiber content provided higher peak strength in the same proportion for 28 curing days compared with 7 curing days. Therefore, it is concluded that both PP and steel fiber provided similar increases in peak strength at the same volume content for 7 and 28 curing days. However, for 3 curing days, the steel fiber needed a stronger and more rigid matrix to be mobilized.

### 3.4. Ductility Index

**Figure 9:**
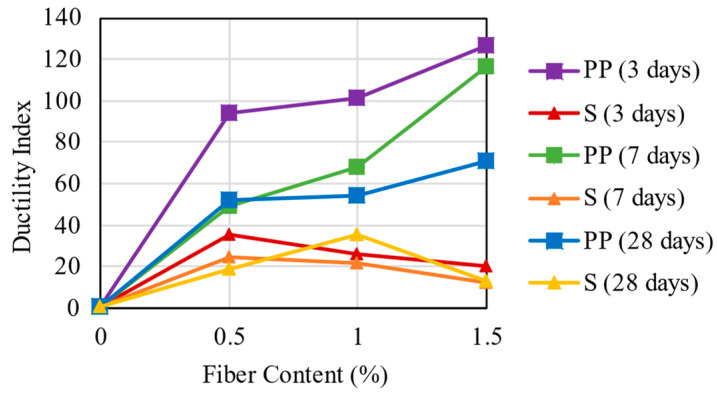
Ductility Index Results for the mixtures.

The results calculated from the ductility index are shown in Figure 9. These responses point out that the mixtures with PP fibers have a higher ductility index (DI) than those with steel fibers due to the higher MOR deflection achieved in these mixtures. The PP fiber mixtures reached DI values of 93–126 for 3 days, 49–116 for 7 days, and 51–71 for 28 days. These findings are different from Jamsawang et al. [7], which reported that PP fibers react to load slower than steel fibers and the values tend to decrease, and these increase with increasing fiber contents. There are two tendencies concerning the PP fiber inclusion in the mixture: the increase in fiber content increases DI values, and the increase in curing time reduces DI values.

The steel fiber inclusion provides DI values of 20–35 for 3 days, 12–24 for 7 days, and 13–35 for 28 days. Excluding the steel fiber mixtures at 1.0% for 28 days of curing time, two tendencies are observed: first is that the increase in fiber content decreases DI values, and the second is that the curing time also reduces DI values. Similar results are reported by Jamsawang et al. [7], in which the steel fiber has a low DI due to its high stiffness, allowing it to be quite effective in carrying the peak load at a small deflection.

### 3.5. Residual Strength

**Figure 10:**
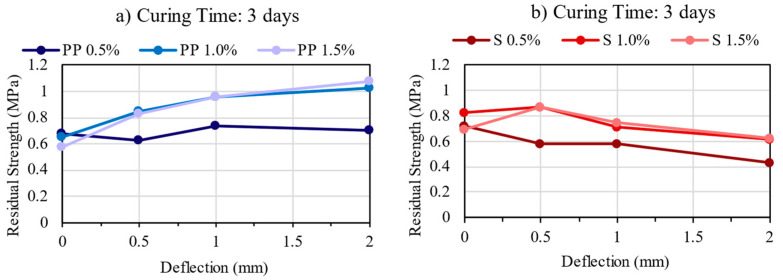
Residual Strength Results for the mixtures: (**a**) PP—3 days; (**b**) Steel—3 days.

**Figure 11:**
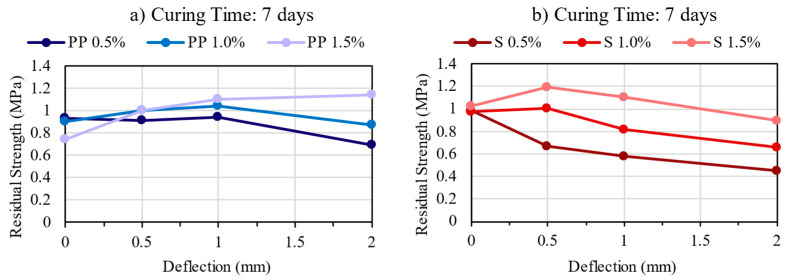
Residual Strength Results for the mixtures: (**a**) PP—7 days; (**b**) Steel—7 days.

**Figure 12:**
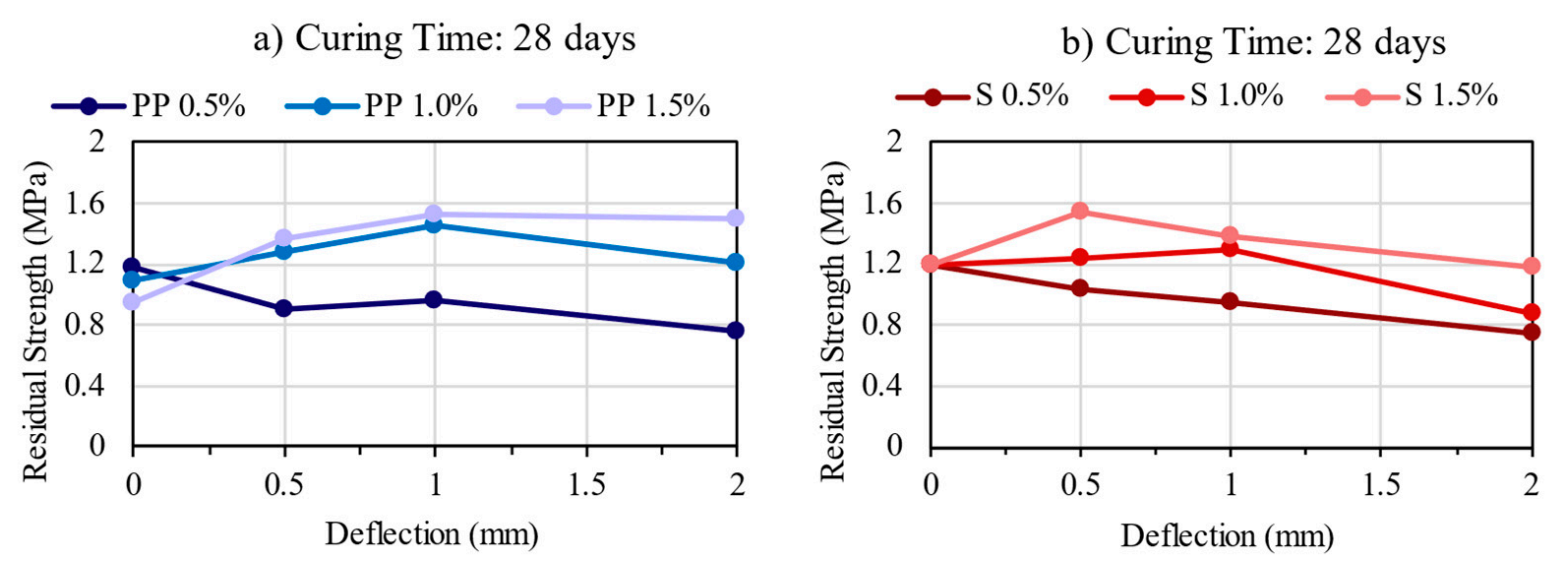
Residual Strength Results for the mixtures: (**a**) PP—28 days; (**b**) Steel—28 days.

The residual strength represents the ability of fiber-reinforced concrete to sustain load after the first crack at different specific deflections [12]. The results are presented in Figure 10, Figure 11 and Figure 12. The residual strength is one of the main benefits of CSR, as the fiber bridging effect helps control the energy release rate. Thus, CSR retains its ability to carry load after the peak (residual load) [12].

Comparing PP and steel fibers, though they have similar values, they do not show the same tendency. In general, it can be observed that the mixtures with PP fibers were able to maintain or increase residual strength with increased deflection, even though the first peak strength was lower than those with steel fibers. In contrast, the steel fibers decrease the residual load after large deflection, but they can maintain or even increase the residual strength at lower deflection values. Thus, for both fibers, it can be verified that increasing the fiber content also increases the residual strength, showing that the fiber content of 1.5% presented the maximum residual strength values.

### 3.6. Flexural Static Modulus

**Figure 13:**
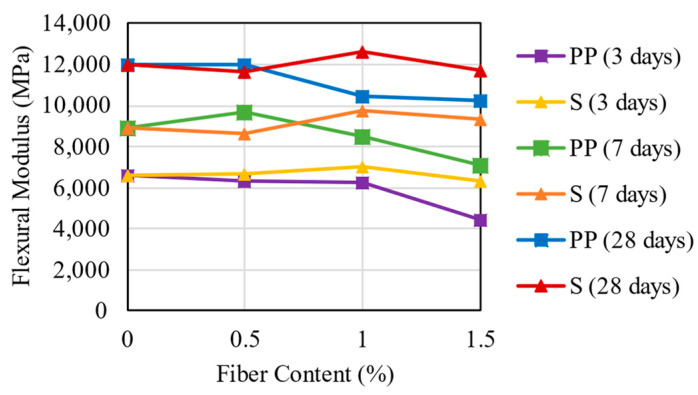
Flexural Modulus Results for the mixtures.

Figure 13 exhibits the flexural modulus values obtained for the PP and steel fibers mixtures. According to the data, the PP fibers had a slight decrease in the flexural modulus compared to the CS specimen. Higher fiber content exhibited a more significant decrease in flexural modulus for the PP fibers. Thus, at 3 days of curing time, PP fiber presented the highest decrease in flexural modulus values of 4.8, 6.1, and 33.2%, respectively, at 0.5, 1.0, and 1.5% fiber content. Therefore, as the CSF matrix becomes more rigid (28 curing days), the PP fiber decreasing effect on flexural modulus is reduced to values varying from 12 to 15% at fiber contents of 1.0 and 1.5%, respectively.

On the contrary, considering the 1.0% fiber content, the steel fiber provided a slight increase in flexural modulus of approximately 4, 5, and 10%, respectively, at 3, 7, and 28 curing days. However, in general, there were no significant differences or trends in flexural modulus for the CSF with steel fibers when compared to the CS samples.

Comparing these results with the polymer-treated fiber cement reported by Onyejekwe and Ghataora [23], a similar trend is observed as the increase in curing time increases the flexural modulus, and the PP fiber results were the ones with less strength since it tends toward a formation of surfaces of weakness at the fiber–soil interface, leading to a reduction in flexural strength. The stiffness evaluation based on the flexural modulus of a cemented base material is an essential component to pavement design and mechanistic analysis. The cemented base materials absorb most of the traffic load; the higher the stiffness, the higher the tensile stresses in the base. These results suggest that PP fibers in excess (1.5%) provide a layer that absorbs fewer loads concerning the lower stiffness, which prevents the base from fatigue cracking but can also overload the other layers.

### 3.7. Toughness

**Figure 14:**
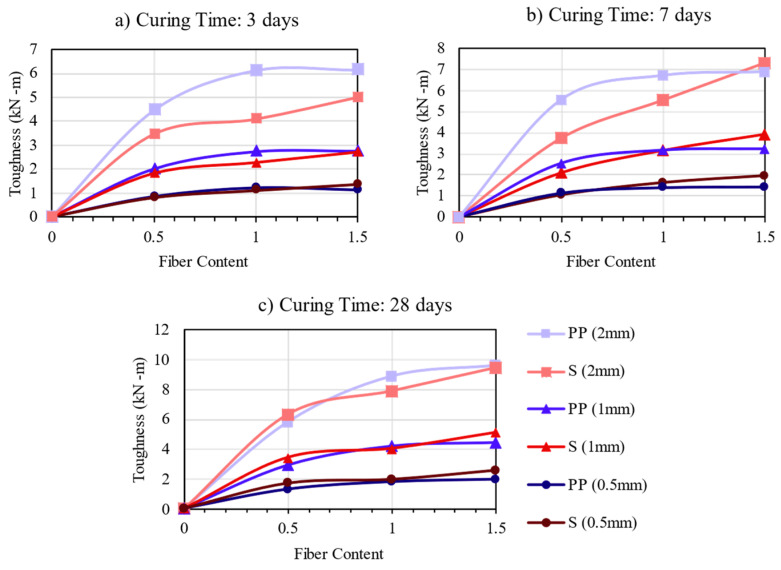
Toughness Results for the mixtures: (**a**) 3 days; (**b**) 7 days; (**c**) 28 days.

The toughness is based on the area below the graphs of the load–deflection curves corresponding to L/150, L/300, and L/600. Figure 14a–c presents the toughness values with the fiber content increment for the mixtures with PP and steel fibers.

The results show that the toughness depends upon fiber content, type, and curing time. Similar to this study, Kim et al. [27] and Jamsawang et al. [6] reported that all CSF had higher toughness than the CS, and fiber contents increased the toughness of both fibers. Thus, this study showed that increasing the curing time did not exhibit differences for L/150 and L/300, but it increased the toughness values for high deflection values. CSF samples that exhibited deflection-hardening behavior performed better than those exhibiting deflection-softening because they absorbed more energy after cracking [6,27].

Different from what is reported by Jamsawang et al. [7], at 3 and 7 days of curing time, the PP and steel fibers had similar values of toughness for 0.5 and 1.0 mm. Thus, at 2.0 mm, the PP fibers had better performance when compared to steel fibers. However, after 28 days of curing time, the results started to be similar to those reported by Jamsawang et al. [7], where steel fibers’ toughness values in high deflection (2.0 mm) significantly increased and reached values near the PP fibers. This can be explained, according to data, that more rigid matrix steel fibers can absorb energy and have higher contributions to toughness. In contrast, the PP fiber mixtures seem to reach an equilibrium with an optimum fiber content of 1.5% and adding values beyond 1.5% may result in no significant increase in toughness. However, this trend is not observed for the steel fiber mixtures, which continue to increase strength beyond the fiber content increment. This explanation is only based on the analyzed data; further investigations are needed.

### 3.8. Effect of the Fiber Inclusion on Mode of Failure

**Figure 15:**
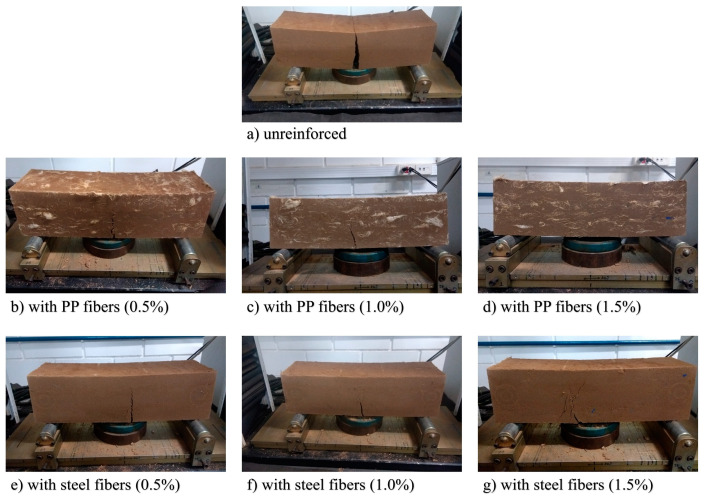
Cracking Patterns Results in elements: (**a**) unreinforced; (**b**–**d**) reinforced with different rate of PP fibers; (**e**–**g**) reinforced with different rate of steel fibers.

To conclude the analysis, the crack patterns are one of the main parameters for characterizing the performance of a fiber type [7,23]. The images from Figure 15 show the mode of failure verified for the CS and CSF mixtures for 28 curing days after the loading procedure. Firstly, all specimens fail due to the tension at the bottom of the tested beam, and all the cracks propagate from bottom to the top. The unreinforced specimen fails with a failure plane at approximately the midspan. On the contrary, it is observed that all reinforced specimens controlled the crack formation, avoiding the brittle behavior that occurs in the unreinforced sample.

The second consideration is that the specimen’s cracking pattern is associated with the fiber content of the sample; the higher the content, the smaller the width and the length of the cracks for both fibers. The result images also confirm the contribution of each fiber in changing the flexural behavior from deflection-softening, as shown in Figure 15 at 0.5% fiber content, or deflection-hardening, as can be seen in Figure 15 at 1.5% fiber content.

Finally, it is observed that, for 1.5% of fiber content, the PP fibers had the prevalence of multiple cracks instead of one single crack. On the contrary, the steel fibers had a well-defined crack. The load–deflection curves of the specimens can explain this fact: while PP fiber mixtures have higher MOR deflections, suggesting they are near the multiple crack curve segment, the steel fibers have lower MOR deflections and are already in the declining curve segment. These observations showed that the steel fibers effectively contribute to the crack after the macrocrack is already formed, redistributing the stress through the fiber matrix [6,7].

## 4. Conclusions

This study investigated the influence of fiber on the flexural performance of a clayed sand soil stabilized with 6% cement for base pavement application. Macro steel and micro polypropylene fibers were blended in the mixtures at 0.5, 1.0, and 1.5% of volume content to evaluate the flexural performance on three different curing periods. Based on the experimental results, the following conclusions are drawn:

- Both fibers could control cracking propagation in the CS, avoiding the bending beam being divided into two parts. Adding fiber and the matrix becoming more rigid with the curing time changed the material behavior from brittle to deflection-hardening for both fibers. For this CS combination, 1.5% of fiber content for both fibers were necessary for a complete deflection-hardening behavior and a prevalence of smaller cracks.
- The LOP strength and flexural modulus were not influenced by the inclusion of steel fiber, which is reasonable since the matrix is intact and, consequently, there is no relative movement between the soil-cement particles and the fiber. There is a loss in the LOP strength and flexural modulus by the PP fiber inclusion in the matrix due to compaction energy loss when inserting PP fibers in the matrix and the low adhesion of PP fiber in the soil-cement.
- PP fiber inclusion provided higher MOR deflection than steel fiber. It is a characteristic that must be considered in pavement because large deformations may not represent a safe structure. Furthermore, the PP fiber mixtures presented higher peak strength for 3 days, confirming that the flexible fibers have a higher contribution in a matrix with lower strength and stiffness and that steel fibers need a more robust matrix to be mobilized.
- Concerning ductility and residual strength, mixtures with PP fibers could retain the loads after cracking more than steel fibers, avoiding cracks formation. However, these contributions were significant for higher deflections, which may not entirely be exploited in a pavement base due to deformation limits.
- In toughness performance, PP fiber addition had, in general, higher increases for 2 mm of deflection for 3 and 7 days of curing time, while steel fiber significantly increased toughness for 28 days of curing time. Both fiber types had higher toughness values with the increase in fiber content. According to the tendencies observed, the contribution of PP fiber in toughness reached a limit for the matrix, while steel fibers may still have larger values by adding fiber content to the matrix.

In summary, the flexural tests confirm the influence of steel fibers on the properties of a cemented matrix, promoting gains in peak strength at small deflections without changing the initial strength and stiffness. The matrix being more rigid implies an increase in this contribution, and there are also benefits to its use concerning ductility, cracking control, and residual strength in the material. In contrast, the PP fiber mixtures obtained greater ductility index, residual strengths, and cracking control than steel fiber mixtures. The increase in deflection highlights the PP fiber contributions. However, as the fiber content increases, the PP fiber presents lower initial strength and flexural modulus. Moreover, the increase in curing time decreases the ratio of the PP fiber contributions, indicating that this type of fiber had better performance in a matrix with lower strength and stiffness. On the other hand, steel fibers need a more robust matrix to be mobilized.

## Figures and Tables

**Table 1:** Main geotechnical properties of the soil.

Property	Value
Liquid Limit	16
Sand (%)	85
Silt (%)	2
Clay (%)	13
Soil classification (unified soil classification system)	SC
Specific gravity	2.646
Maximum dry unit weight from standard compaction test (kN/m^3^)	19.83
Optimum moisture content from standard compaction test	9.24%

**Table 2:** Main physical and mechanical fiber properties (from the manufacturers).

Type	Micro Polypropylene	Macro Steel
Length (mm)	20	33
Designation	PP	SF
Shape	Straight	Hooked
Section	Circular	Circular
Diameter of width	12 μm	0.75 mm
Specific weight (g/cm^3^)	0.91	7.80
Tensile Strength (MPa)	865	1100
Young’s modulus (GPa)	9	210

## Data Availability

The data presented in this study are available on request from the corresponding author.

## References

[1] Maaitah O.N. (2012). Soil Stabilization by Chemical Agent. Geotech. Geol. Eng..

[2] Khattak M.J., Alrashidi M. (2006). Durability and mechanistic characteristics of fiber reinforced soil–cement mixtures. Int. J. Pavement Eng..

[3] Consoli N.C., de Moraes R.R., Festugato L. (2011). Split tensile strength of monofilament polypropylene fiber-reinforced cemented sandy soils. Geosynth. Int..

[4] Ban H., Park S.-W. (2014). Characteristics of modified soil-aggregate system and their application in pavements. KSCE J. Civ. Eng..

[5] Al-Aghbari M.Y., Mohamedzein Y.E.-A., Taha R. (2009). Stabilisation of desert sands using cement and cement dust. Proc. Inst. Civ. Eng. Ground Improv..

[6] Jamsawang P., Voottipruex P., Horpibulsuk S. (2015). Flexural Strength Characteristics of Compacted Cement-Polypropylene Fiber Sand. J. Mater. Civ. Eng..

[7] Jamsawang P., Suansomjeen T., Sukontasukkul P., Jongpradist P., Bergado D.T. (2018). Comparative flexural performance of compacted cement-fiber-sand. Geotext. Geomembr..

[8] Sobhan K. (2008). Improving the Tensile Strength and Toughness of a Soil-Cement-Fly Ash Pavement Subgrade with Recycled HDPE Strips. GeoCongress 2008.

[9] McGOWN A., Andrawes K.Z., Al-Hasani M.M. (1978). Effect of inclusion properties on the behaviour of sand. Geotechnique.

[10] Festugato L., da Silva A.P., Diambra A., Consoli N.C., Ibraim E. (2018). Modelling tensile/compressive strength ratio of fibre reinforced cemented soils. Geotext. Geomembr..

[11] Olgun M. (2013). Effects of polypropylene fiber inclusion on the strength and volume change characteristics of cement-fly ash stabilized clay soil. Geosynth. Int..

[12] Sukontasukkul P., Jamsawang P. (2012). Use of steel and polypropylene fibers to improve flexural performance of deep soil–cement column. Constr. Build. Mater..

[13] Tang C.-S., Shi B., Zhao L.-Z. (2010). Interfacial shear strength of fiber reinforced soil. Geotext. Geomembr..

[14] Yi X.W., Ma G.W., Fourie A. (2015). Compressive behaviour of fibre-reinforced cemented paste backfill. Geotext. Geomembr..

[15] (2019). Standard Test Method for Flexural Strength of Soil-Cement Using Simple Beam with Third-Point Loading.

[16] Brasse K., Tracz T., Zdeb T. (2020). The Effect of the Type and Amount of Synthetic Fibers on the Effectiveness of Dispersed Reinforcement in Soil-Cements. Materials.

[17] Li C. (2005). Mechanical Response of Fiber-Reinforced Soil. Master’s Thesis.

[18] Hejazi S.M., Sheikhzadeh M., Abtahi S.M., Zadhoush A. (2011). A simple review of soil reinforcement by using natural and synthetic fibers. Constr. Build. Mater..

[19] De Paula T.M., Consoli N.C., Festugato L., Favretto F., DaRonco J.V.L. (2019). Behaviour of fibre-reinforced cemented sand under flexural tensile stress. E3S Web Conf..

[20] Viswanadham B.V.S., Jha B.K., Pawar S.N. (2010). Influence of geofibers on the flexural behavior of compacted soil beams. Geosynth. Int..

[21] Liang L., Xu Y., Hu S. (2022). Bending and Crack Evolution Behaviors of Cemented Soil Reinforced with Surface Modified PVA Fiber. Materials.

[22] Zhang P., Li Q., Wei H. (2010). Investigation of Flexural Properties of Cement-Stabilized Macadam Reinforced with Polypropylene Fiber. J. Mater. Civ. Eng..

[23] Onyejekwe S., Ghataora G.S. (2014). Effect of Fiber Inclusions on Flexural Strength of Soils Treated with Nontraditional Additives. J. Mater. Civ. Eng..

[24] Zhang J., Xu W., Gao P., Yao Z., Su L., Qiu N., Huang W. (2022). Compressive strength characteristics of hybrid fiber-reinforced cemented soil. Int. J. Pavement Eng..

[25] Zabielska-Adamska K., Dobrzycki P., Wasil M. (2023). Estimation of Stiffness of Non-Cohesive Soil in Natural State and Improved by Fiber and/or Cement Addition under Different Load Conditions. Materials.

[26] Oliveira P.J.V., Correia A.A.S., Teles J.M.N.P.C., Custódio D.G. (2016). Effect of fibre type on the compressive and tensile strength of a soft soil chemically stabilised. Geosynth. Int..

[27] Kim D.J., Park S.H., Ryu G.S., Koh K.T. (2011). Comparative flexural behavior of Hybrid Ultra High Performance Fiber Reinforced Concrete with different macro fibers. Constr. Build. Mater..

[28] Festugato L., Menger E., Benezra F., Kipper E.A., Consoli N.C. (2017). Fibre-reinforced cemented soils compressive and tensile strength assessment as a function of filament length. Geotext. Geomembr..

[29] Ayeldeen M., Kitazume M. (2017). Using fiber and liquid polymer to improve the behaviour of cement-stabilized soft clay. Geotext. Geomembr..

[30] Consoli N.C., Bassani M.A.A., Festugato L. (2010). Effect of fiber-reinforcement on the strength of cemented soils. Geotext. Geomembr..

[31] Fatahi B., Khabbaz H., Fatahi B. (2012). Mechanical characteristics of soft clay treated with fibre and cement. Geosynth. Int..

[32] Cristelo N., Cunha V.M.C.F., Dias M., Gomes A.T., Miranda T., Araújo N. (2015). Influence of discrete fibre reinforcement on the uniaxial compression response and seismic wave velocity of a cement-stabilised sandy-clay. Geotext. Geomembr..

[33] Sharma R.K. (2017). Laboratory study on stabilization of clayey soil with cement kiln dust and fiber. Geotech. Geol. Eng..

[34] Tran K.Q., Satomi T., Takahashi H. (2018). Improvement of mechanical behavior of cemented soil reinforced with waste cornsilk fibers. Constr. Build. Mater..

[35] Anggraini V., Asadi A., Huat B.B.K., Nahazanan H. (2015). Effects of coir fibers on tensile and compressive strength of lime treated soft soil. Measurement.

[36] Mandal T., Tinjum J.M., Gokce A., Edil T.B. (2016). Protocol for Testing Flexural Strength, Flexural Modulus, and Fatigue Failure of Cementitiously Stabilized Materials Using Third-Point Flexural Beam Tests. Geotech. Test. J..

[37] Zhang P., Wei X.H. (2011). Study on Flexural Strength and Flexural Modulus of Elasticity of Cement Stabilized Aggregate. Advanced Materials Research.

[38] Chindaprasirt P., Jamsawang P., Sukontasukkul P., Jongpradist P., Likitlersuang S. (2021). Comparative mechanical performances of cement-treated sand reinforced with fiber for road and pavement applications. Transp. Geotech..

[39] Fedrigo W., Visser A.T., Steyn W.J.M., Núñez W.P. (2021). Flexural behaviour of lightly cement stabilised materials: South Africa and Brazil. Road Mater. Pavement Des..

[40] Nematollahi B., Sanjayan J., Shaikh F.U.A. (2014). Comparative deflection hardening behavior of short fiber reinforced geopolymer composites. Constr. Build. Mater..

[41] (2022). Standard Specification for Portland Cement.

[42] (2012). Soil-Cement—Mixture for Use in Pavement Layer—Procedure.

[43] Cristelo N., Cunha V.M.C.F., Gomes A.T., Araújo N., Miranda T., Lopes M.D.L. (2017). Influence of fibre reinforcement on the post-cracking behaviour of a cement-stabilised sandy-clay subjected to indirect tensile stress. Constr. Build. Mater..

[44] (2019). Standard Test Method for Flexural Performance of Fiber-Reinforced Concrete (Using Beam with Third-Point Loading).

[45] Sobhan K., Mashnad M. (2002). Tensile Strength and Toughness of Soil–Cement–Fly-Ash Composite Reinforced with Recycled High-Density Polyethylene Strips. J. Mater. Civ. Eng..

[46] Sukontasukkul P., Pomchiengpin W., Songpiriyakij S. (2010). Post-crack (or post-peak) flexural response and toughness of fiber reinforced concrete after exposure to high temperature. Constr. Build. Mater..

